# *Colletotrichum siamense* Strain LVY 9 Causing Spot Anthracnose on Winterberry Holly in China

**DOI:** 10.3390/microorganisms11040976

**Published:** 2023-04-09

**Authors:** Lin Feng, Yahui Zhang, Weiliang Chen, Bizeng Mao

**Affiliations:** Ministry of Agriculture Key Lab of Molecular Biology of Crop Pathogens and Insects, Institute of Biotechnology, College of Agriculture & Biotechnology, Zhejiang University, Hangzhou 310058, China

**Keywords:** winterberry holly, anthracnose, *Colletotrichum siamense*, pathogenicity, multilocus phylogeny

## Abstract

Winterberry holly (*Ilex verticillata*) is an economically valuable landscaping ornamental plant. Serious outbreaks have been reported, in its leaf tips curl upward, irregular black brown spots appear on leaves, and extensive defoliation is commonly observed. The incidence in Hangzhou was estimated at 50% and resulted in large economic losses for growers in 2018. Samples were collected from the main cultivation area in Zhejiang Province. In total, 11 fungal isolates were obtained from diseased leaves through a single-spore purification method, and isolate LVY 9 exhibited strong pathogenicity. Based on morphology and molecular phylogenetic analyses based on multilocus sequence typing of the glyceraldehyde-3-phosphate dehydrogenase (*GAPDH*), internal transcribed spacer (*ITS*) regions, actin (*ACT*), calmodulin (*CAL*), and chitin synthase (*CHS-1*) genes, we identified the pathogen as *Colletotrichum siamense*, causative agent of anthracnose of winterberry holly.

## 1. Introduction

Winterberry holly (*Ilex verticillata*) is a deciduous shrub that is especially valued for its masses of glossy, bright red berries in winter. Also known as Black Alder, its branches are often used for decoration during the holiday season [1]. Winterberry holly is widely cultivated in Europe, the United States, and other countries for its unique ornamental qualities and high adaptability. To date, there are more than 30 varieties cultivated and sold in Europe and America, such as “Oosterwijk”, “Winter Red”, “Berry heavy”, and “A. Gray”, among many others [2,3].

In China, in addition to their desirable floral qualities and broad marketability, especially in the Spring Festival [4]. The vivid red berries symbolize good luck and auspicious harvest. The cultivation area of winterberry holly has been rapidly expanding since 2006 in Zhejiang, Shandong, Fujian, Henan, Jilin, and Gansu provinces, as well as in other regions of China [3,5].

Fruit rot of deciduous holly (Ilex spp. L.) was recently reported as an emerging threat in nurseries in the mid-western and eastern USA [6]. Disease symptoms including early defoliation and withered or rotten fruit were observed throughout all of the fruit development and maturation period [7]. Previous work has indicated that multiple fungal pathogens such as *Alternaria alternata*, *Diaporthe ilicicola*, as well as other minor pathogens such as *A. arborescens*, *Botryosphaeriaceae*, *Colletotrichum fioriniae*, *C.nymphaeae*, *D.eres*, and *Epicoccum nigrum* can cause primary and/or secondary infections during the growing season through inoculation by wind, rain splash, and pollinators [8,9]. To date, there have been very few reports describing diseases of winterberry holly, so the relevant disease resistance mechanisms remain unclear.

In 2018, growers reported the occurrence of a new anthracnose disease of winterberry holly in Zhejiang province, China, with primary symptoms including black or brown leaves, irregular leaf spots, and sparse white mycelium. The incidence of diseased leaves ranged from 10% to 50%, severely affecting the aesthetic quality. It is of great economic significance determine the pathogenic agent underlying this disease so that a timely and effective management strategy can be deployed. Here, in this work, we isolate the causative agent of this new disease, and identify it as *Colletotrichum siamense*.

## 2. Materials and Methods

### 2.1. Sample Collection and Pathogen Isolation

**Figure 1:**
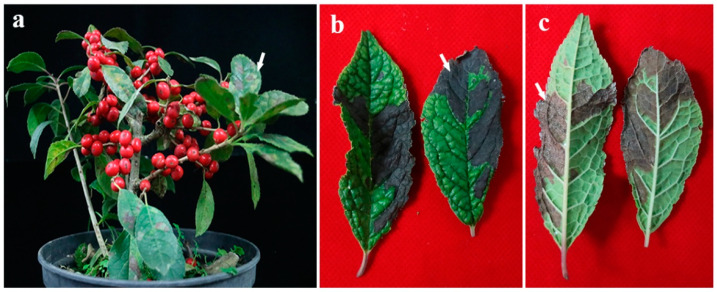
Symptoms of diseased winterberry holly. (**a**) Symptomatic leaves change from green to yellowish green, and gradually become grayish brown Coalesced irregular leaf spots eventually resulted in early plant defoliation. The arrow points to the spot. (**b**,**c**) A large area of irregular black–brown leaf spots with dense white mycelia on winterberry holly’s defoliation. The arrow points to white mycelia.

The symptomatic samples were collected from Zhejiang province in 2018 (Figure 1). Diseased leave tissues were surface-sterilized with 75% ethanol for 30 s, and then 0.1% mercuric chloride solution for 3 min, rinsed five times by sterile distilled water, and air dried; the axenic tissues were subsequently cut into about 25 mm^2^ pieces and plated on potato dextrose agar (PDA) medium containing 0.2 mg/mL^−1^ ampicillin sodium. The plates were incubated at 28 °C in the dark for 7–14 days. Pure mycelia were obtained by hyphae tip separation and stored in PDA slants covered by paroline at 4 °C [10,11].

### 2.2. Pathogenicity Test

Two inoculation methods were used for the pathogenicity assay. The vitro leaf method was described by Lin and Dissanayake [8,9,12]. One-month-old healthy leaves were collected from potted plants (A.Gray), surface-sterilized with 75% ethanol and immersed in sterile water-Tween 80 solutions (0.3% *v*/*v*) 3 times before inoculation. About 5 mm-diameter mycelial plugs were picked up from the culture medium and placed on left and right sides of the same leaf, then those leaves were incubated in a 10 cm^2^ petri dish containing a wet sterile filter paper, inoculated with sterile PDA as controls. There were 3 replicates of each isolate and the experiment was repeated three times. The dishes were placed in a growth chamber under artificial light (12/12 h light/dark) at 28 °C for 7 days. The second inoculation technique was described by Weir [13]: 1 × 10^6^ conidia/mL of the spore suspensions were prepared in sterile distilled water. Leaves were wounded evenly by the sterilized needle, and then the front and back of the leaves of the biennial plants were sprayed with the suspensions. Sterile distilled water was used as control. Then, all plants were maintained in a greenhouse under 100% relative humidity at temperatures between 30 °C (day) and 28 °C (night) with natural photoperiods. The pathogen was re-isolated from leaf spots, and its identity confirmed by morphological characteristics, which is consistent with the Koch’s postulates.

### 2.3. Morphological Characteristics

Mycelia discs (7 mm in diameter) from 7-day-old PDA cultures were sub-cultured on PDA medium in incubator, at 28 °C. The mycelium growth was measured every day for 7 consecutive days, and the colony features (size and color) were recorded after 7 days [14]. The mycelia were removed from the surface of the plate to enhance the sporulation under a mixture of fluorescent white and UV light with 12 h dark at 20 °C for 15–20 days [13,15,16]. The conidia were examined visually and microscopically for morphological features, the mycelia (7 mm diameter) with conidia were observed in SEM (Hitachi Model SU-8010) [17,18,19].

Appressorium was produced using a slide culture technique [13]. A mycelium colony on PDA with 1 cm2 was placed in an empty petri dish (9 cm) and immediately covered with a sterile cover slip, then transferred into an empty square petri dish (10 cm) containing sterile water. After 14 days, the cover slip was removed, and a drop of lactic acid was added on a glass slide [13,14].

### 2.4. Phylogenetic Analysis

#### 2.4.1. Genomic DNA Extraction and PCR Amplification

Genomic DNA was extracted using Ezup Column Fungi Genomic DNA Purification Kit (Sangon Biotech (Shanghai, China) Co., Ltd.) following the manufacturer’s instructions. The PCR reaction mixture contained 9 μL of sterile water, 13 μL of 2 × PCR Master Mix (TSINGKE, Beijing, China), 1 μL of each primer (10 μM), 1μL of genomic DNA. PCR was conducted in Applied Biosystems LongGene Thermal Cycler (LongGene Scientific Instruments (Hangzhou, China) Co., Ltd.) under the following conditions for the ITS: 94 °C for 5 min; 35 cycles of denaturation at 94 °C for 30 s, annealing at 52 °C for 30 s, and extension at 72 °C for 1 min; and then followed by a final extension step at 72 °C for 10 min. Amplification programs for the other genes were the same except the annealing temperatures: *ACT* (58 °C ), *CAL* (59 °C ), *CHS-1* (58 °C ), *GAPDH* (60 °C). The primers and references are summarized in Table 1.

#### 2.4.2. Fungal Isolates Phylogenetic Analysis

The obtained sequences were edited by BioEdit 7.1.3.0 [24] and multiple alignments were generated with MAFFT 7.273 [25]. To construct the phylogenetic tree, available data for species of Colletotrichum were downloaded from the GenBank database (Table A1). Maximum likelihood (ML) analyses were performed using IQ-TREE [26,27] with the concatenation of the ACT, CAL, CHS-1, GAPDH, and ITS regions. The best evolutionary model was selected through Model Finder [28], as follows: TIM2 + F + R4 model for ACT, TIM + F + R3 for CAL, TIM + F + R3 for CHS-1, HKY + F + R2 for GAPDH and TIM2 + F + R3 for ITS. Confidence of the branch points was evaluated using 5000 bootstrap replicates. The ML values equal to or above 75% values were shown on a tree for significantly supported nodes. Bayesian Inference (BI) was applied to generate a phylogeny tree by MrBayes ver.3.2.6 [29]. GTR + F + I + G4 model was used to the analyses of the gene of ACT, CAL, CHS1, ITS and the HKY + F + G4 model for GAPDH following Ronquist and Huelsenbeck [29] for BI analyses with 200,000 replicates and the phylogenetic tree was sampled every 100 generations. The first 500 trees were removed for the burn-in phase. The consensus of the remaining trees was constructed with resulting node frequencies that were treated as Bayesian posterior probabilities (PP). BI posterior probability (BI-PP) values equal to or above 0.95 were determined to be significant. The tree was rooted with the outgroup, *Monilochaetes infuscans*.

## 3. Results

### 3.1. The Strain LVY 9 Was Pathogen of Anthracnose on Winterberry Holly through Koch’s Postulates

**Figure 2:**
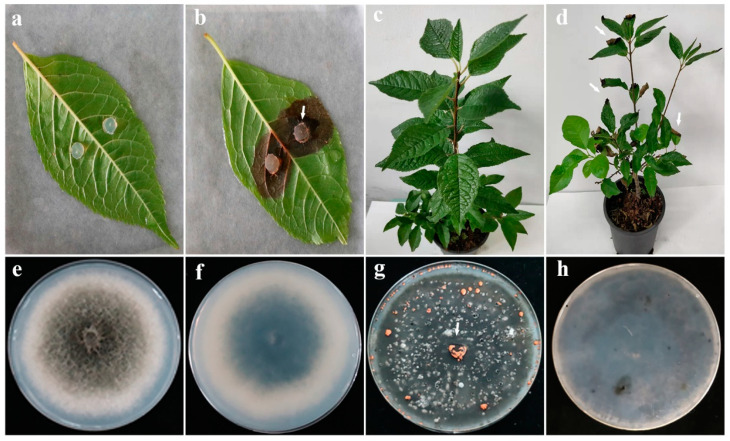
Symptoms of winterberry holly leaves after incubation with isolate LVY 9. In vitro inoculation, brown necrotic lesions with bright orange conidial masses appear (**b**) compared with control (**a**) after 7 days. The arrow points to orange conidia (**b**). In spray inoculation, grayish brown spots appear on tips of leaves with obvious curly phenotype compared with control (**c**) after 30 days. The arrow points to irregular spots (**d**). The mycelia are grayish brown with white edge (**e**) and reserve is turquoise (**d**) after strain LVY 9 on PDA for 7 d. Orange masses of conidia released from pycnidia after strain LVY 9 under a mixture of fluorescent white and UV light on PDA 20 d (**g**,**h**). The arrow points to orange conidia (**g**). Strain LVY 9 resembles *Colletotrichum gloeosporioides* by SEM and light microscopy.

We identified and isolated eleven isolates (LVY 1–11) from symptomatic leaves of *Ilexverticillata* (Hangzhou city, Zhejiang province, China) based on morphological features and confirmation by Sanger sequencing. These isolates belonged to genera *Colletotrichum, Alternaria, Botryosphaeria, Leptosphaeria, Cercospora,* and *Emericella,* and subsequent pathogenicity tests indicated that only inoculation with *Colletotrichum* (isolate LVY 9) resulted in symptom formation on leaves in vitro. These symptoms included brown necrotic lesions with dense whitish-grey aerial mycelia, and a few bright orange conidial masses near the point of inoculation at 7 days post infection (dpi) (Figure 2a,b). The mycelia are grayish brown with white edge and reserve is turquoise after strain LVY 9 on PDA for 7 days. Colonies were round, swell, neat edge, with cottony radial growth of white. In particular, large clusters of bright orange conidia were produced on PDA for 30 d. These characteristics are very similar to those of *Colletotrichum gloeosporioides* complex. Following spray inoculations, similar symptoms appeared at 30 dpi in winterberry nursery fields (Figure 2c). In diseased plants, leaf tips of inoculated plants exhibited an obvious curly phenotype (Figure 2d). In addition, we observed irregular gray black spots on the leaves. We were able to successfully re-isolate the *Colletotrichum* isolates from these diseased plants, thereby fulfilling Koch’s postulates.

**Figure 3:**
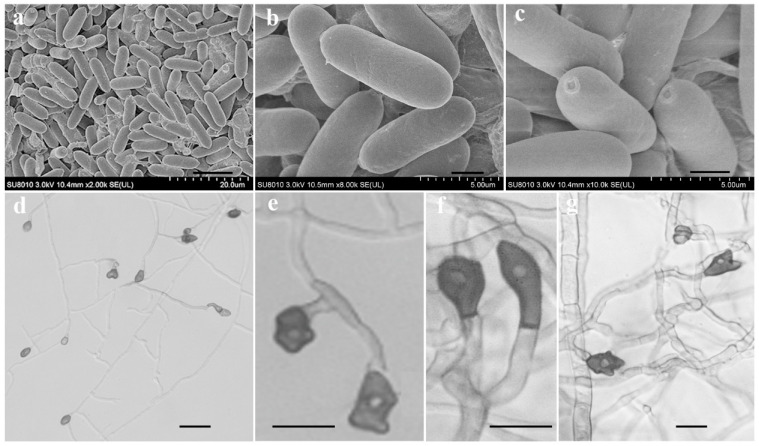
Conidia and appressorium characteristics of isolate LVY 9 on PDA. Conidia are smooth-walled, hyaline, and cylindrical, with obtuse to slightly rounded ends (**a**–**c**). Bars = 10, 2, 1 µm, respectively; appressoria appear brown to dark brown, ovoid, clavate, and slightly irregular in shape(**d**–**g**). Bars = 20, 10, 10, 5 µm, respectively.

To characterize the microscopic features of the pathogen, in addition to its symptoms and colony morphology, we selected a representative isolate for morphological characterization by SEM and light microscopy. On PDA medium, isolate LVY 9 colonies first appeared white, then became gray to dark grey, with an average growth rate of approximately 10.72 mm/day. Bright orange conidia and conidiophores were produced under continuous UV + fluorescent white lights with a 12:12 h light: dark cycle (Figure 2g). Scanning electron microscopy revealed that conidial dimensions were 7.8–12.7 × 2. 6–3.6 µm, and conidia were smooth-walled, hyaline, and cylindrical, with obtuse to slightly rounded ends (Figure 3a–c). Under light microscopy, appressoria (5.8–10.7 × 3.9–7.7 µm) were observed to form mostly from mycelium on slide cultures, and appeared brown to dark brown, ovoid, clavate, and slightly irregular in shape (Figure 3d–h). Morphological features of the isolate LVY 9 were highly similar to those of species belonging to the *Colletotrichum gloeosporioides* complex [13,30,31,32].

### 3.2. Strain LVY 9 Was Identified as Colletotrichum siamense by Phylogenetic Analyses

**Figure 4:**
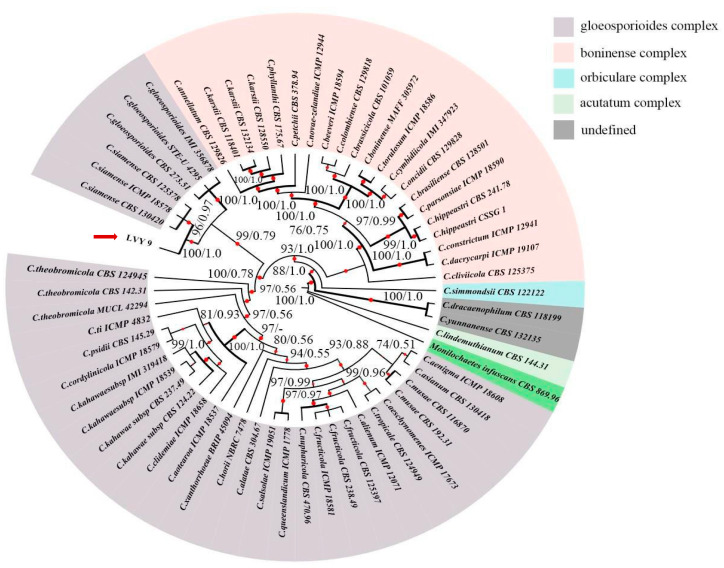
Maximum likelihood (ML) tree generated from the combined ACT, CAL, CHS-1, GAPDH and ITS sequence data of 59 taxa of *Colletotrichum*, of which 21 are in *C.boninense* complex, 34 are in *C.gloeosporioides* complex, 1 is in *C.orbiculare* complex, 1 is in *C.acutatum* complex, 2 are undefined. Clades with >75% ML_BS (left) and 0.50 BI-PP (right) are indicated by the corresponding support values. Parsimony and likelihood bootstrap support values ≥ 95% are indicated at the nodes and branches with Bayesian posterior probabilities above 0.95 given in bold. The ex-type strains are in bold. Dashes indicate support values lower than 75% ML-BS and 0.50 BI-PP. The tree is rooted with *Monilochaetes infuscans* CBS 869.96. The red arrow points to strain LVY9.

For rigorous molecular identification, we used partial sequences from *ACT* (GenBank number: OQ652092), *CAL* (GenBank number: OQ652091), *GAPDH* (GenBank number: OQ652089), *CHS-1* (GenBank number: OQ652090)*,* and *ITS* (GenBank number: OQ651128) from isolate LVY9 for species level identification of the pathogen (see Table 1 for primers and source studies). Maximum likelihood-based phylogenetic reconstruction of these concatenated sequences (2370 nucleotides) including *ACT* (1–311 bp), *CAL* (312–1119 bp), *CHS-1* (1120–1420 bp), *GADPH* (1421–1736 bp), and *ITS* (1737–2370 bp) was congruent with that of a Bayesian inference (BI) tree for the five concatenated loci. Relationships among almost all of the reference isolates could be clearly distinguished at the species level. Furthermore, phylogenetic analysis indicated that isolate LVY 9 clustered with *C. siamense* CBS 130,420, *C. siamense* ICMP 18,587, and *C. siamense* CBS 125,378, thus forming a distinct clade, which was highly supported in both ML-BS (100%) and BI-PP (1.0) models (Figure 4). Based on these findings, we concluded that the LVY9 isolate was a strain of *C. siamense*, the previously reported causative agent of anthracnose in winterberry holly.

## 4. Discussion

The asexual genus *Colletotrichum* is largely comprised of economically and agriculturally destructive plant pathogens [33], causing major losses in yield and productivity to a wide range of fruit, vegetable and ornamental crops such as strawberry, mango, chilli, pear, eggplant, cowpea, mandevilla and rhododendron [18,34,35,36,37,38]. Based on morphological studies and phylogenetic analyses using actin (*ACT*), calmodulin (*CAL*), chitin synthase 1 (*CHS-1*), glyceraldehyde-3-phosphate dehydrogenase (*GAPDH*), internal transcribed spacers (*ITS*), and β-Tubulin 2 (*TUB2*), Damm and colleagues [15] effectively separated the *C. acutatum* species complex into 30 species, while Weir and coworkers [13] distinguished 22 species and one subspecies within the *C. gloeosporioides* species complex. In particular, *C. siamense* was reported to cause anthracnose on persea americana, pistacia vera, coffea arabica, vitis vinifera, malus domestica, and hymenocallis Americana [13]. Chunhua indicated that the 13 isolates of *C. gloeosporioides* species complex from the rubber tree in Hainan Province were identified as *C.siamense* and *C. fructicola* [39]. Simlarly, *C. siamense* was involved in walnut, pyrus spp., litchi pepper, amorphophallus konjac, photinia × fraseri in China [40,41,42,43,44]. In this work, we first report the presence of *C. siamense,* the causative pathogen for anthracnose of the ornamental plant winterberry holly, in China.

In the *Colletotrichum gloeosporioides* complex, *C. gloeosporioides*, *C. siamense,* and *C. fructicola* exhibit similar morphological characteristics including conidial size, shape, and appressoria formation. *C. siamense* is genetically close to C. *fructicola*, but *C*. *fructicola* has slightly longer and narrower cylindrical or subcylindrical conidia that have irregularly shaped, crenate, brown to dark brown appressoria, and branched hyphae [36,45]. However, the conidia of *C. siamense* are fusiform with obtuse to slightly rounded ends, occasionally oblong and ovoid. Previous reports also confirm that this species exhibits regular- to slightly irregular-shaped appressoria [14]. In contrast, *C. gloeosporioides* conidia appear cylindrical, although slightly tapered with obtuse or slightly rounded to oblong ends. The *C. gloeosporioides* appressoria are circular to slightly irregular, thus differing from those of *C. siamense* [14,46]. In this study, the shapes of the conidia and appressoria of isolates matched those of *C. siamense*, described by Prihastuti [14]. Moreover, the size of appressoria was similar to that of *C. siamense*, although the conidia described by Sharma [36,47] were smaller. Mycelial growth rates of isolates obtained in this study were also slower than those of the strain of *C. siamense* described by Yaowen [48]. We speculated that these morphological differences may be due (at least in part) to responses to the environment, growth media, and host.

Since morphological identification is important but not definitive for *Colletotrichum*, sequence analysis and infection behavior are also used to discriminate down to species level. As mentioned in previous studies, species in the *C. gleosporioide* species complex are genetically distinct from those in the *C. boninense* complex, but have highly similar micro-morphologiesm [13,15,16,49]. Initial sequence analyses relying on ITS were unable to satisfactorily distinguish among *Colletotrichum* species due to their high level of evolutionary conservation. In this work, we therefore used several genes to identify the LVY9 strain that were previously reported to successfully resolve *Colletotrichum* species [18,50]. Notably, *ITS* can separate *C. gloeosporioides* from all other *Colletotrichum* species, but cannot reliably separate *C. siamense* from *C. alienum*, *C. fructicola*, or *C. tropicale*. These species are best distinguished using *CAL* or *TUB2* [13]. The causative agent of leaf spots on *Sterculia nobilis* in China was identified as *C. siamense* using a combination of *ITS*, *ACT*, *GAPDH*, *CAL*, *CHS-1,* and *TUB2* genes [48]. In this study, we used a combinations of five genes (*ACT*, *CAL*, *CHS-1*, *GAPDH* and *ITS*) to identify the isolates obtained from winterberry holly, thus providing strong molecular evidence for the identification of the isolates as *C. siamense.*

*C. siamense* was originally described as a pathogen of coffee berries in Thailand [14]. This species has since been confirmed to infect more than 60 plant species worldwide [13,15,16]. This paper presents the first report describing *C. siamense* as the causal agent of anthracnose of winterberry holly in Zhejiang, China. As winterberry holly is commonly used as a bonsai or cut flower, the disease directly reduces its ornamental value. In addition, the occurrence of disease may harm the introduction and cultivation of winterberry holly. This identification of the disease-causing species facilitates the establishment of control measures, not only for winterberry holly, but for all of the known hosts of this pathogen. This study also provides a basis for future studies of the molecular mechanisms of this pathogenic interaction, identification of disease-resistant varieties, and creation of stable resistant materials.

## 5. Conclusions

According to Koch’s rule, The strain LVY9 was elected as the pathogen causing anthracnose of Winterberry Holly in Zhejiang Province, China. The strain LVY9 was identified as the *C. gloeosporioides* complex by observing the morphology features of mycelia, conidia and appressorium. The strain LVY9 was identified as *C. siamense* by further analysis of the phylogenetic tree that combined Actin, Calmodulin, Glyceraldehyde-3- Phosphate dehydrogenase, Chitin synthase and Internal transcribed spacer genes. So, we concluded that *C. siamense* was causative agent of anthracnose in winterberry holly.

## Figures and Tables

**Table 1:** Primers used in this study, with sequences and sources.

Gene	Product Name	Primers	Sequence (5′–3′)	Reference
*ACT*	Actin	ACT-512F ACT-783R	ATGTGCAAGGCCGGTTTCGC TACGAGTCCTTCTGGCCCAT	[20]
*CAL*	Calmodulin	CL1C CL2C	GAATTCAAGGAGGCCTTCTC CTTCTGCATCATGAGCTGGAC	[13]
*GAPDH*	Glyceraldehyde-3- Phosphate dehydrogenase	GDF GDR	GCCGTCAACGACCCCTTCATTGA GGGTGGAGTCGTACTTGAGCATGT	[21]
*CHS-1*	Chitin synthase	CHS-79F CHS-345R	TGGGGCAAGGATGCTTGGAAGAAG TGGAAGAACCATCTGTGAGAGTTG	[20]
*ITS*	Internal transcribed spacer	ITS-1F ITS-4	CTTGGTCATTTAGAGGAAGTAA TCCTCCGCTTATTGATATGC	[22] [23]

## Data Availability

Not applicable.

## Appendix A

**Table A1:** GenBank accession numbers of the accepted Colletotrichum species.

Colletotrichum Species	Culture Collection	GenBank Accession Number					References
		ITS	GAPDH	CAL	ACT	CHS-1	
C.aenigma	ICMP 18,608 *	JX010244	JX010044	JX009683	JX009443	JX009774	[13]
C.aeschynomenes	ICMP 17,673 * ATCC 201,874	JX010176	JX009930	JX009721	JX009483	JX009799	[13]
C.alatae	CBS 304.67 * ICMP 17,919	JX010190	JX009990	JX009738	JX009483	JX009799	[13]
C.alienum	ICMP 12,071 *	JX010251	JX010028	JX009654	JX009572	JX009882	[13]
C.annellatum	CBS 129,826 CH1 *	JQ005222	JQ005309	JQ005743	JQ005570	JQ005396	[16]
C.aotearoa	ICMP 18,537 *	JX010205	JX010005	JX009611	JX009564	JX009853	[13]
C.asianum	ICMP 18,580 * CBS 130,418	FJ972612	JX010053	FJ917506	JX009584	JX009867	[13]
C.beeveri	ICMP 18,594 * CBS 125,827	JQ005171	JQ005258	JQ005692	JQ005519	JQ005345	[16]
C.boninense	MAFF 305,972 * CBS 123,755	JQ005153	JQ005240	JQ005674	JQ005501	JQ005327	[13]
C.brasiliense	CBS 128,501 PAS12 *	JQ005235	JQ005322	JQ005756	JQ005583	JQ005409	[16]
C.brassicicola	CBS 101,059 LYN 16,331 *	JQ005172	JQ005259	JQ005693	JQ005520	JQ005346	[16]
C.clidemiae	ICMP 18,658 *	JX010265	JX009989	JX009645	JX009537	JX009877	[13]
C.cliviae	CBS 125,375 *	GQ485607	GQ856756	GQ849464	GQ856777	GQ856722	[17]
C.colombiense	CBS 129,818 G2 *	JQ005174	JQ005261	JQ005695	JQ005522	JQ005348	[16]
C.constrictum	ICMP 12,941 * CBS 128,504	JQ005238	JQ005325	JQ005759	JQ005586	JQ005412	[16]
C.cordylinicola	ICMP 18,579 MFLUCC 090,551 *	JX010226	JX009975	HM470237	HM470234	JX009864	[13]
C.cymbidiicola	IMI 347,923 *	JQ005166	JQ005253	JQ005687	JQ005514	JQ005340	[16]
C.dacrycarpi	ICMP 19,107 * CBS 130,241	JQ005236	JQ005323	JQ005757	JQ005584	JQ005410	[16]
C.dracaenophilum	CBS 118,199 *	JX519222	JX546707	-	JX519238	JX519230	[17]
C.fructicola	CBS 125,397 * ICMP 18,646	JX010173	JX010032	JX009674	JX009581	JX009874	[13]
C.fructicola	CBS 238.49 * ICMP 17,921	JX010181	JX009923	JX009671	JX009495	JX009839	[13]
C.fructicola	ICMP 18,581 * CBS 130,416	JX010165	JX010033	FJ917508	FJ907426	JX009866	[13]
C.gloeosporioides	CBS 273.51 * ICMP 19,121	JX010148	JX010054	JX009745	JX009558	JX009903	[13]
C.gloeosporioides	STE-U4295 * CBS 112,999	JQ005152	JQ005239	JQ005673	JQ005500	JQ005326	[16]
C.gloeosporioides	IMI 356,878 * ICMP 17,821 CBS 112,999	JX010152	JX010056	JX009731	JX009531	JX009818	[13]
C.hippeastri	CSSG 1 * CBS 125,376	JQ005231	JQ005318	JQ005752	JQ005579	JQ005405	[16]
C.hippeastri	CBS 241.78	JX010293	JX009932	JX009740	JX009485	JX009838	[13]
C.horii	NBRC 7478 * ICMP 10,492	GQ329690	GQ32961	JX009604	JX009438	JX009752	[13]
C.kahawae subsp	CBS 124.22* ICMP 19,122	JX010228	JX009950	JX009744	JX009536	JX009902	[13]
C.kahawae subsp	IMI 319,418 * ICMP 17,816	JX010231	JX010012	JX009642	JX009452	JX009813	[13]
C.kahawae subsp	ICMP 18,539 *	JX010230	JX009966	JX009635	JX009523	JX009800	[13]
C.kahawae subsp	CBS 237.49 * ICMP 17,922	JX010238	JX010042	JX009636	JX009450	JX009840	[13]
C.karstii	CBS 132,134 *	HM585409	HM858391	HM582013	HM581995	HM582023	[16]
C.karstii	CBS 128,550 ICMP 17,896	JQ005219	JQ005306	JQ005740	JQ005567	JQ005393	[16]
C.karstii	CBS 118,401	JQ005192	JQ005279	JQ005713	JQ005540	JQ05366	[16]
C.lindemuthianum	CBS 144.31	JQ005779	JX546712	-	JQ005842	JQ005800	[17]
C.musae	CBS 192.31 ICMP 17,923	JX010143	JX009929	JX009690	JX009587	JX009841	[13]
C.musae	CBS 116,870 * ICMP 19,119	JX010145	JX010047	JX009687	JX009551	JX009849	[13]
C.novae-zelandiae	ICMP 12,944 * CBS 128,505	JQ005228	JQ005315	JQ005749	JQ005576	JQ005402	[16]
C.nupharicola	CBS 470.96 * ICMP 18,187	JX010187	JX009972	JX009663	JX009437	JX009835	[13]
C.oncidii	CBS 129,828 *	JQ005169	JQ005256	JQ005690	JQ005517	JQ005343	[16]
C.parsonsiae	ICMP 18,590 * CBS 128,525	JQ005223	JQ005320	JQ005754	JQ005581	JQ005407	[16]
C.petchii	CBS 378.94 *	JQ005223	JQ005310	JQ005744	JQ005571	JQ005397	[16]
C.phyllanthi	CBS 175.67 MACS 271 *	JQ005221	JQ005308	JQ005742	JQ005569	JQ005395	[16]
C.psidii	CBS 145.29 * ICMP 19,120	JX010219	JX009967	JX009743	JX009515	JX009901	[13]
C.queenslandicum	ICMP 1778 *	JX010276	JX009934	JX009691	JX009447	JX009899	[13]
C.salsolae	ICMP 19,051 *	JX010242	JX009916	JX009696	JX009562	JX009863	[13]
C.siamense	CBS 125,378 * ICMP 18,642	JX010278	JX010019	JX009709	GQ85675	GQ856730	[13]
C.siamense	CBS 130,420 * ICMP 19,118	HM131511	HM13147	JX009713	HM13157	JX009895	[13]
C.siamense	ICMP 18,578 * CBS 130,417	JX010171	JX009924	FJ917505	FJ907423	JX009865	[13]
C.simmondsii	CBS 122,122, BRIP 28,519 *	JQ948276	JQ948606	FJ917510	JQ949588	JQ948937	[16]
C.theobromicola	CBS 124,945 * ICMP 18,649	JX010294	JX010006	JX009591	JX009444	JX009869	[13]
C.theobromicola	CBS 142.31 * ICMP 17,927	JX010286	JX010024	JX009582	JX009516	JX009830	[13]
C.theobromicola	MUCL 42,294 * ICMP 17,957	JX010289	JX009962	JX009597	JX009575	JX009821	[13]
C.ti	ICMP 4832 *	JX010269	JX009952	JX009649	JX009520	JX009898	[17]
C.torulosum	ICMP 18,586 * CBS 128,544	JQ005164	JQ005251	JQ005685	GU27899	GU228291	[17]
C.tropicale	CBS 124,949 * ICMP 18,653	JX010264	JX010007	JX009719	JX009489	JX009870	[13]
C.xanthorrhoeae	BRIP 45,094 * ICMP 17,903	JX010261	JX009927	JX009653	JX009478	JX009823	[13]
C.yunnanense	CBS 132,135 *	JX546804	JX546706	-	JX519239	JX519231	[17]

Note: * refers to ex-type strains of *Colletotrichum* species
